# Changes in oxygen uptake in patients with non-ischemic dilated cardiomyopathy and left bundle branch block following left bundle branch area pacing

**DOI:** 10.3389/fcvm.2025.1551551

**Published:** 2025-09-02

**Authors:** Guillermo Gutiérrez-Ballesteros, Francisco Mazuelos-Bellido, José López-Aguilera, Manuel Crespín-Crespín, Rafael González-Manzanares, María Asunción García-Merino, Dolores Mesa-Rubio, Miguel Romero-Moreno, Manuel Pan Álvarez-Osorio, José María Segura Saint-Gerons

**Affiliations:** ^1^ Cardiology Department, Reina Sofia University Hospital, Córdoba, Spain; ^2^ Maimonides Institute for Research in Biomedicine of Córdoba (IMIBIC), Córdoba, Spain

**Keywords:** cardiopulmonary exercise test, dilated cardiomyopathy, left bundle branch block, left bundle branch area pacing, oxygen uptake

## Abstract

**Introduction and objectives:**

Left bundle branch area pacing (LBBAP) has been associated with good clinical and echocardiographic outcomes and seems to be an alternative to conventional resynchronization therapy. However, data regarding functional outcomes are scarce. Our objective was to evaluate, using cardiopulmonary exercise testing (CET), changes in the functional capacity of patients with an indication for cardiac resynchronization therapy after LBBAP.

**Methods:**

We conducted a prospective analysis of a cohort of patients with non-ischaemic dilated cardiomyopathy (NIDCM), left bundle branch block, QRS duration >130 ms, New York Heart Association functional class (NYHA-FC) II–IV, and left ventricular ejection fraction (LVEF) < 40% who underwent LBBAP. CET was performed before the procedure and after 6 months of follow-up. The primary endpoint was the change in peak oxygen uptake (VO_2_). The secondary endpoints included evaluation of clinical, echocardiographic, analytical, and other CET parameters.

**Results:**

A total of 50 patients were included (44% female, 64 ± 11 years, LVEF 28 ± 7%). At baseline, peak VO_2_ was 15.4 ± 4.9 ml/kg/min, and VO_2_ at the first ventilatory threshold was 10.5 ± 2.9 ml/kg/min. At follow-up, we observed an increase of 3 ml/kg/min (95% CI 1.7–4.4; *p* < 0.01) and 2.6 ml/kg/min (95% CI 1.6–3.5; *p* < 0.01), respectively. Independent predictors of peak VO_2_ at follow-up were baseline peak VO_2_ and baseline QRS duration. Improvement was observed in the remaining CET, echocardiography, and clinical parameters.

**Conclusions:**

In symptomatic patients with non-ischaemic dilated cardiomyopathy, LVEF < 40%, and left bundle branch block, LBBAP was associated with an improvement in peak VO_2_. Baseline QRS duration and baseline peak VO_2_ were independent predictors of this parameter at follow-up.

## Introduction

Left bundle branch area pacing (LBBAP) is a physiological pacing technique that has replaced His bundle pacing (HBP) at many centers due to its lower implantation complexity and more efficient pacing parameters (1, 2). The success rate of implantation is high at >80% in patients with an indication for cardiac resynchronization therapy (CRT) and >90% with an indication for bradycardia (3). In patients with left bundle branch block (LBBB) and indication for CRT (4), LBBAP has shown positive clinical and echocardiographic results, both in observational registries and in comparative studies compared with CRT with coronary sinus pacing (CRT-CS) (5–8), so it may be an alternative to CRT-CS.

However, to date, the functional response assessed by cardiopulmonary exercise testing (CET) has not been analyzed in patients with an indication for CRT and LBBB after LBBAP. CET provides an objective analysis of patients' functional capacity and can mitigate a possible placebo effect after device implantation; in addition, the parameters assessed during the test have a significant prognostic impact, irrespective of the left ventricular ejection fraction (LVEF) (9, 10).

This study aimed to assess functional capacity through changes in O_2_ uptake (VO_2_) values in CET after implantation of an LBBAP device in patients with non-ischaemic dilated cardiomyopathy (NIDCM), LVEF <40% [according to the definition of heart failure (HF) with reduced LVEF], LBBB, QRS duration >130 ms, and New York Heart Association functional class (NYHA-FC) II–IV after at least 3 months of optimal medical therapy (OMT) and with 6 months of follow-up (4).

## Materials and methods

### Study design and population

This was an observational, prospective, and single-center study in which a cohort of patients undergoing LBBAP was enrolled consecutively. The *inclusion criteria* were (I) age > 18 years; (II) NIDCM with LVEF < 40% with no associated coronary disease (ruled out with coronarography or coronary computed tomography angiography); (III) NYHA-FC II–IV despite OMT up to maximum tolerated doses for at least 3 months (4); and (IV) QRS duration > 130 ms with LBBB morphology. The *exclusion criteria* were (I) inability to perform CET on a treadmill; (II) pregnancy; (III) atrial fibrillation (AF) with poorly controlled ventricular response (average heart rate > 110 bpm on 24-hour Holter); (IV) dilated cardiomyopathy (DCM) secondary to chronic right ventricular (RV) pacing; (V) severe primary valve disease; (VI) previous acute myocardial infarction; (VII) frequent premature ventricular contractions (PVC) (>10% PVC on 24-hour Holter) previous to LBBAP; (VIII) history of drug abuse, including alcohol; and (IX) life expectancy < 1 year at enrollment. We explained the purpose of the study to the patients who met all the inclusion criteria and none of the exclusion criteria, and they signed the informed consent prior to participating in the study. Throughout the study, recruited patients were not allowed to participate in the cardiac rehabilitation program, so if they had not completed it before inclusion, they would be included once the follow-up was completed; the use of new drugs for heart failure was not permitted during the duration of the study if they were not previously taking them; however, during follow-up, the dose of previously used drugs could be increased if tolerance improved after LBBAP**.** The project was approved on 23 February 2022 by the local Ethics Committee in accordance with the Declaration of Helsinki and the guidelines of the International Conference for Good Clinical Practice (Committee reference 5293).

### Study objectives

The primary objective was to assess changes in peak VO_2_ in the study population 6 months after LBBAP device implantation. The secondary endpoints analyzed were (A) *predictors of peak VO_2_ in follow-up*; (B) *changes in other CET parameters*, (I) % of predicted peak VO_2_, (II) exercise time (mm:ss), (III) VO_2_ at first ventilatory threshold (VT1) (ml/kg/min) and time to reach VT1 (mm:ss), (IV) peak oxygen (O_2_) pulse (ml/beat), (V) ventilatory efficiency (VE/VCO slope), (VI) oxygen uptake efficiency slope (OUES), (VII) ventilatory class, (VIII) respiratory exchange ratio (RER); (C) *echocardiographic data*, (I) LVEF (%), (II) mitral regurgitation (MR) (grade I–IV), (III) ventricular volumes (ml/m^2^); (D) *analytical parameters*, (I) NT-ProBNP (pg/ml); (E) *clinical parameters*, (I) NYHA-FC, (II) patients' quality of life using the Kansas City Cardiomyopathy Questionnaire-12 (KCCQ-12), (III) distance walked in meters in the 6-min walk test (6MWT); and (F) *variables related to the implant and follow-up of the LBBAP device*.

### Baseline assessment

Prior to implantation, all patients underwent a clinical evaluation, a transthoracic echocardiogram (TTE), and a treadmill CET (ergometer, Schiller MTM-1400; gas analyzer, Ganshorn PowerCube® Diffusion +). Cardiac magnetic resonance imaging (MRI) was not part of the baseline assessment within the study protocol but was recommended for young patients (<60 years).

At the baseline visit, the following assessments were performed: medical history, blood test, 6MWT, KCCQ-12 test, TTE, and CET. In TTE, we assessed LVEF using the Simpson's biplane method and end-systolic (ESV) and end-diastolic (EDV) volumes of the left ventricle (LV) as well as the severity of MR according to current recommendations (11). The CET was performed and analyzed by a cardiologist expert in this field (JL-A or MC-C), an individualized treadmill protocol was used for each patient in percentage of slope and maximum velocity to perform a test that *a priori* lasted between 8 and 12 min; before starting, adequate calibration of the gas analyzer was confirmed and spirometry was performed. CET was stopped when patients developed limiting dyspnea or complex ventricular arrhythmias. We considered CET maximal if we observed a RER > 1.1 at maximum effort or >1.09 after 2 min of recovery. We defined peak VO_2_ as the highest VO_2_ value in the last 30 s of exercise and expressed it adjusted for weight both in absolute value (ml/kg/min) and as a percentage relative to age, sex, weight, height and the exercise protocol used according to the Wasserman equation [% predicted peak VO_2_ (% ppVO_2_)]. VO_2_ at VT1 was obtained by combining the ventilatory equivalents method and the V-slope method. In addition, we collected data on peak O_2_ pulse (ml/beat), the VE/VCO_2_ slope, OUES, and changes in ventilatory classification according to previously published methods (9, 10, 12).

### Implantation and follow-up of the LBBAP device

We used LBBAP as the first choice of CRT in all patients with no previous attempts at HBP or CS pacing. The technique used has been previously described, although a simplified approach was used (13). The implant was performed with the C315 double-curved sheath (Medtronic, Inc., Minneapolis, MN, USA) and SelectSecure^™^ lead (model 3830, 69 cm; Medtronic, Inc., Minneapolis, MN, USA); in cases where implantation was initially unsuccessful, we used the SL10 septal-curved sheath (Medtronic, Inc., Minneapolis, MN, USA). After vascular access, once the sheath had been positioned in the right atrium (RA), in the right anterior oblique projection (20°–30°), the sheath was moved toward the right ventricle (RV) and oriented toward the interventricular septum, and a lower position was sought at the junction of the proximal third of the tricuspid valve with the two distal thirds of the RV. Subsequently, in unipolar configuration, pacing was carried out from the tip of the lead, checking for the following parameters on the electrocardiogram (ECG): (I) “W” morphology in the QRS in lead V1, (II) an amplitude in lead II greater than that in III and (III) AVL positivity with negative AVR. If none of these criteria were observed, a proximal area was sought by modifying the angulation and direction of the sheath; if this was not achieved after multiple attempts, an anatomical approach was used. A left anterior oblique projection (20°–30°) position was then used to confirm that the lead and sheath were located coaxially with respect to the interventricular septum and proceeded to apply clockwise torque to the ventricular lead to move it through the interventricular septum until it reached the left bundle branch area, checking the QRS morphology every 4–5 turns and using the fixation beats as a guide (14). To monitor the implant and for interval measurement, we used the LabSystem Pro™ v4.1 polygraph (Boston Scientific, Charlestown, MA, USA; bipolar filter, 10–100 Hz; electrocardiogram filter, 0.1–25 Hz; sweep speed, 100 mm/s).

Based on the QRS morphology and the electrical parameters obtained, the patients were divided into three groups (15):

1. *Left bundle branch pacing (LBBP).* QRS paced in a unipolar configuration shows right bundle branch block (RBBB) morphology and any of the following parameters:
  • Interval between pacing artifact and peak R wave in V6 (LVAT-V6) <100 ms.
  • Interval between peak R wave in V6 and peak R wave in V1 > 33 ms (V6-V1 interpeak interval).
  • Transition from non-selective LBBP (NS-LBBP) capture to selective capture (S-LBBP) with constant LVAT-V6 or transition from NS-LBBP to left ventricular septal pacing (LVSP) with LVAT-V6 prolongation ≥15 ms.
2. *Left ventricular septal pacing (LVSP).* Paced QRS in unipolar configuration shows RBBB morphology that does not meet LBBP criteria.
3. *Deep septal pacing (DSP).* Paced QRS shows “W” morphology or “QS” pattern in lead V1.

The procedure was considered successful if LBBP or LVSP was obtained, both of which fall under the term LBBAP. The procedure was considered a failure if DSP was obtained. Concomitant implantation of an implantable cardioverter defibrillator (ICD) during the procedure was performed depending on the patient's age, comorbidities, and the presence of late gadolinium enhancement (LGE) in cardiac MRI (if cardiac MRI was performed) (16, 17).

After the procedure, the AV interval of the device was adjusted to achieve complete capture of the left bundle branch because adjustment of the AV interval to facilitate fusion with the patient's intrinsic beat may be altered in situations of high adrenergic tone, where conduction through the atrioventricular node is enhanced, with the consequent loss of left bundle branch area capture.

The following data were collected on the implant: (I) procedure time, (II) fluoroscopy time, (III) procedure success, (IV) acute complications, (V) type of device implanted, (VI) threshold, (VII) R-wave detection, and (VIII) impedance. The following measurements were taken after implantation with a sweep speed of 100 mm/s: (I) baseline and paced QRS duration (ms) measured from the first to the last deflection on the 12-lead electrocardiogram, (II) V6-V1 interpeak interval (ms), and (III) LVAT-V6 (ms).

### Follow-up

Device follow-up was performed 2–3 weeks after implantation to assess complications and analyze electrical parameters and ventricular pacing (VP) percentage, and if this was <90%, the appropriate treatment was indicated: adjustment of the AV interval, treatment with antiarrhythmic drugs or ablation in the event of a new appearance of high density PVC (>10%), and AV node ablation in permanent AF or pulmonary vein ablation in paroxysmal or persistent AF. Six months after device implant, clinical and device follow-ups were performed, and the blood test, 6MWT, KCCQ-12, TTE, and CET were repeated, using the same study protocol as in the baseline CET.

### Sample size

The sample size was calculated using the following assumptions: bilateral hypothesis testing, alpha 0.05, power 90%, and an estimated mean basal peak VO_2_ of 14 ml/kg/min (18, 19) with a standard deviation (SD) of 4 ml/kg/min, we used as reference basal peak VO_2_ of the patients included in the Miracle and Contak CD studies where the mean peak VO_2_ at baseline were between 13.5 and 14 ml/kg/min (18, 19). Based on this, the enrolment of 49 subjects with ventricular dysfunction and LBBB would allow the detection of significant differences between pre- and postimplantation peak VO_2_ of at least 2 ml/kg/min. The estimated proportion of losses in follow-up was 10%.

### Statistical analysis

Normal distribution of variables was assessed with the Kolmogorov–Smirnov test. Quantitative variables are shown as mean ± standard deviation (SD) or median and interquartile range (IQR) according to whether or not they follow a normal distribution. Categorical variables are shown as absolute values and percentages. For the comparison of quantitative variables, Student's *t*-test for paired data or the Wilcoxon test was used as appropriate; differences between quantitative variables are expressed as means or medians with 95% confidence intervals. Categorical variables were compared with Pearson's *χ*^2^ test, Fisher's exact test, or McNemar's test. Variables associated with peak VO_2_ at 6 months were analyzed using multivariate linear regression. In the initial multivariate model, peak VO_2_ at 6 months was included as a dependent variable, and clinically relevant variables based on previous studies and those with a *p* < 0.150 in the univariate models were included as independent variables. The presence of multicollinearity was analyzed, and the least influential variables were excluded using the backward elimination method. For statistical analysis, SPSS Statistics version 25.0 (IBM, Chicago, IL, USA) and R software (version 4.3.2; R Foundation for Statistical Computing, Austria) were used. Differences with a *p* < 0.05 were considered statistically significant.

## Results

### Baseline characteristics

Between March 2022 and March 2024, a total of 50 patients with a mean age of 64 ± 11 years were enrolled; 22 (44%) were female, and all of them completed follow-up. Thirty-nine (78%) patients were in NYHA-FC II and 11 (22%) in NYHA-FC III–IV, and they had an adequate OMT [47 (94%) patients with beta-blockers, 49 (98%) with ARNI/ACEI/ARB, 37 (74%) with SGLT-2 inhibitors, and 44 (88%) with mineralocorticoid receptor antagonists]. At the time of inclusion, 47 (94%) patients were in sinus rhythm, and baseline QRS had a median duration of 176 ms (IQR 156–183). In TTE, the mean LVEF prior to LBBAP was 28% ± 7%, and 12 (24%) patients had mitral regurgitation of at least moderate grade (grade II–IV). The baseline 6MWT distance was 393 ± 80 m, the KCCQ-12 score was 42 points (IQR 35–58), and the NT-ProBNP value was 1,075 pg/ml (IQR 485–1999). Baseline cardiac MRI was performed in 16 patients, with LGE being present in 2 of them (Table 1).

**Table 1:** Baseline characteristics of patients.^a^

	Patients (*N* = 50)
Sex (female)	22 (44%)
Age (years)	64 ± 11
Cardiovascular risk factors, *N* (%)	
Hypertension	25 (50%)
Diabetes	13 (26%)
Hyperlipidemia	15 (30%)
Atrial fibrillation	
No	41 (82%)
Paroxysmal	6 (12%)
Persistent/permanent	3 (6%)
BMI (kg/m^2^)	29 ± 12
Medical treatment, *N* (%)	
BB	47 (94%)
ARNI/ACEI/ARB	49 (98%)
ARNI	44 (88%)
ACEI/ARB	5 (10%)
SGLT2i	37 (74%)
MRA	44 (88%)
Loop diuretics	28 (56%)
Anticoagulation	9 (18%)
Clinical parameters	
Functional class, *N* (%)	
NYHA II	39 (78%)
NYHA III–IV	11 (22%)
QRS (ms)	176 (156–183)
6MWT distance (m)	393 ± 80
KCCQ-12 score	42 (35–58)
NT-ProBNP (pg/ml)	1,075 (485–1,999)
Hemoglobin (g/dl)	14.5 ± 1.3
Creatinine (mg/dl)	0.96 (0.83–1.1)
Echocardiographic parameters	
LVEF (%)	28 ± 7
LVEDV (ml/m^2^)	99 ± 31
LVESV (ml/m^2^)	73 ± 28
Mitral regurgitation (grade II–IV), *N* (%)	12 (24%)

6MWT, 6-min walk test; ACEI, angiotensin-converting enzyme inhibitors; ARB, angiotensin II receptor blockers; ARNI, angiotensin receptor/neprilysin inhibitor; BB, beta-blockers; BMI, body mass index; KCCQ-12, Kansas City Cardiomyopathy Questionnaire; LVEDV, left ventricular end-diastolic volume; LVEF, left ventricular ejection fraction; LVESV, left ventricular end-systolic volume; MRA, mineralocorticoid receptor antagonist; NYHA, New York Heart Association; SGLT2i, sodium–glucose transport protein 2 inhibitors.

^a^ Quantitative variables are expressed as mean ± standard deviation or as median and interquartile range as appropriate. Qualitative variables are expressed as absolute values and percentages.

### Implantation and follow-up of the device

The median procedure time was 65 min (IQR 46–102) and was successful in 49 (98%) cases, of which LBBP was achieved in 42 (84%) and LVSP in 7 (14%) according to predefined criteria. A dual-chamber device was implanted in 45 (90%) cases, a single-chamber device in 2 (4%), and a three-chamber device (CRT-D) in 3 (6%). After implantation, we observed a median QRS reduction of 36 ms [95% confidence interval (CI) 32–40 ms, *p* < 0.01], the mean LVAT was 81 ± 16 ms, and the V6-V1 interpeak interval was 51 ± 21 ms. The threshold at the end of the procedure was 0.5 V/0.4 ms (IQR 0.5–0.75), R-wave detection was 8.3 mV (IQR 5.6–12), and impedance was 494 Ω (IQR 456–594). After the procedure, we observed one acute dislocation of the LBBAP lead that required reintervention and repositioning. AV node ablation was performed in three patients due to permanent AF with VP < 90% in the first device follow-up. At 6-month follow-up, median VP was 99% (IQR 98–99), and electrical parameters showed no difference in the pacing threshold 0.5 V/0.4 ms (IQR 0.5–0.75) (95% CI −0.06 to 0.05, *p* = 0.66); however, we observed an increase in R-wave to 12.8 mV (8.3–18.5) (+3.05 mV, 95% CI 1.6–4.5, *p* < 0.01) as well as a reduction in LBBAP lead impedance to 399 Ω (361–418) [–123 Ω, 95% CI −161 to –95, *p* < 0.01]. In the 6-month revision, one patient had a threshold increase of 0.75 V/0.4 ms, but no loss of LBBAP was noted, and no intervention was required (Table 2).

**Table 2:** Procedure characteristics and device follow-up.^a^

	Patients (*N* = 50)
Skin-to-skin procedure duration (min)	65 (46–102)
Procedure success, *N* (%)	49 (98%)
LBBAP	
LBBP, *N* (%)	42 (84%)
LVSP, *N* (%)	7 (14%)
Fluoroscopy time (min)	16 ± 13
Type of device	
Single-chamber pacemaker, *N* (%)	2 (4%)
Dual-chamber pacemaker, *N* (%)	45 (90%)
Three-chamber device (CRT-D), *N* (%)	3 (6%)
Basal QRS duration (ms)	176 (156–183)
Paced QRS duration (ms)	136 (125–144)
LVAT-V6 (ms)	81 ± 16
RV_6_–RV_1_ interpeak interval (ms)	51 ± 21
LBBAP lead postimplant electrical parameters.	
Threshold (V/0.4 ms)	0.5 (0.5–0.75)
R-wave amplitude (mV)	8.3 (5.6–12)
Impedance (ohm)	494 (456–594)
Acute procedure complications, *N* (%)	1 (2%)^b^
LBBAP lead electrical parameters at follow-up (6 months)	
Threshold (V/0.4 ms)	0.5 (0.5–0.75)
R-wave amplitude (mV)	12.8 (8.3–18.5)
Impedance (ohm)	399 (361–418)
Ventricular pacing (%)	99 (98–99)
Complications in follow-up, *N* (%)	1 (2%)^c^

CRT-D, cardiac resynchronization therapy defibrillator; LBBAP, left bundle branch area pacing; LBBP, left bundle branch pacing; LVSP, left ventricular septal pacing; RV_6_–RV_1_ interpeak interval, interval (ms) between the peak of the R wave in V6 and the peak of the R wave in V1; LVAT-V6, interval between the pacing spike and the peak of the R wave in V6; ms, milliseconds; mV, millivolts; Ohm, ohms; V, volts.

^a^ Quantitative variables are expressed as mean ± standard deviation or as median and interquartile range as appropriate. Qualitative variables are expressed as absolute values and percentages.

^b^ Dislocation of the left bundle branch pacing lead requiring reintervention.

^c^ Left bundle branch area pacing lead threshold increased by 0.75 V but did not result in loss of left bundle branch capture.

### Changes in cardiopulmonary exercise test parameters

In the baseline CET, the median exercise time was 07:23 min (IQR 05:38–10:25), and the test was considered maximal in 29 (58%) cases. The mean peak VO_2_ was 15.4 ± 4.9 ml/kg/min, which was 69% ± 20% of the %ppVO_2_. VO_2_ at VT1 of 10.5 ± 2.9 ml/kg/min was observed and reached at 03:55 min (IQR 02:40–04:45), O_2_ pulse was 9.6 ± 4.1 ml/beat, OUES value was 1.69 ± 0.69, and VE/VCO_2_ slope was 31.1 (IQR 27.4–34.6). A total of 30 (60%) patients were in ventilatory class II–IV.

All patients completed follow-up. The follow-up CET was considered maximal in 25 (50%) cases (*p* = 0.15 with respect to baseline). Within the primary endpoint, an increase in peak VO_2_ of +3.0 ml/kg/min (95% CI 1.7–4.4, *p* < 0.01) was observed, which corresponded to a 12% increase in % ppVO_2_ (95% CI 5–19, *p* < 0.01) (Central figure). Among the secondary endpoints, we observed an increase in VO_2_ at VT1 of +2.6 ml/kg/min (95% CI 1.6–3.5, *p* < 0.01), a delay in the time to reach VT1 of +01:15 min (95% CI 00:45–02:00, *p* < 0.01), an increase in exercise time of +02:25 min (95% CI 01:25–03:29, *p* < 0.01), an increase in the OUES of +0.21 (95% CI 0.05–0.37, *p* < 0.01), an increase in the O_2_ pulse of +2.6 ml/beat (95% CI 1.5–3.7, *p* < 0.01), and a reduction in the number of patients in ventilatory class II–IV [30 (60%) patients at baseline vs. 17 (34%) patients at follow-up, *p* < 0.01]. Likewise, a significant reduction in the VE/VCO2 slope of −1.7 points [95% CI −3.6 to –0.2, *p* = 0.04] was observed without changes in RER (Table 3).

**Central Figure:**
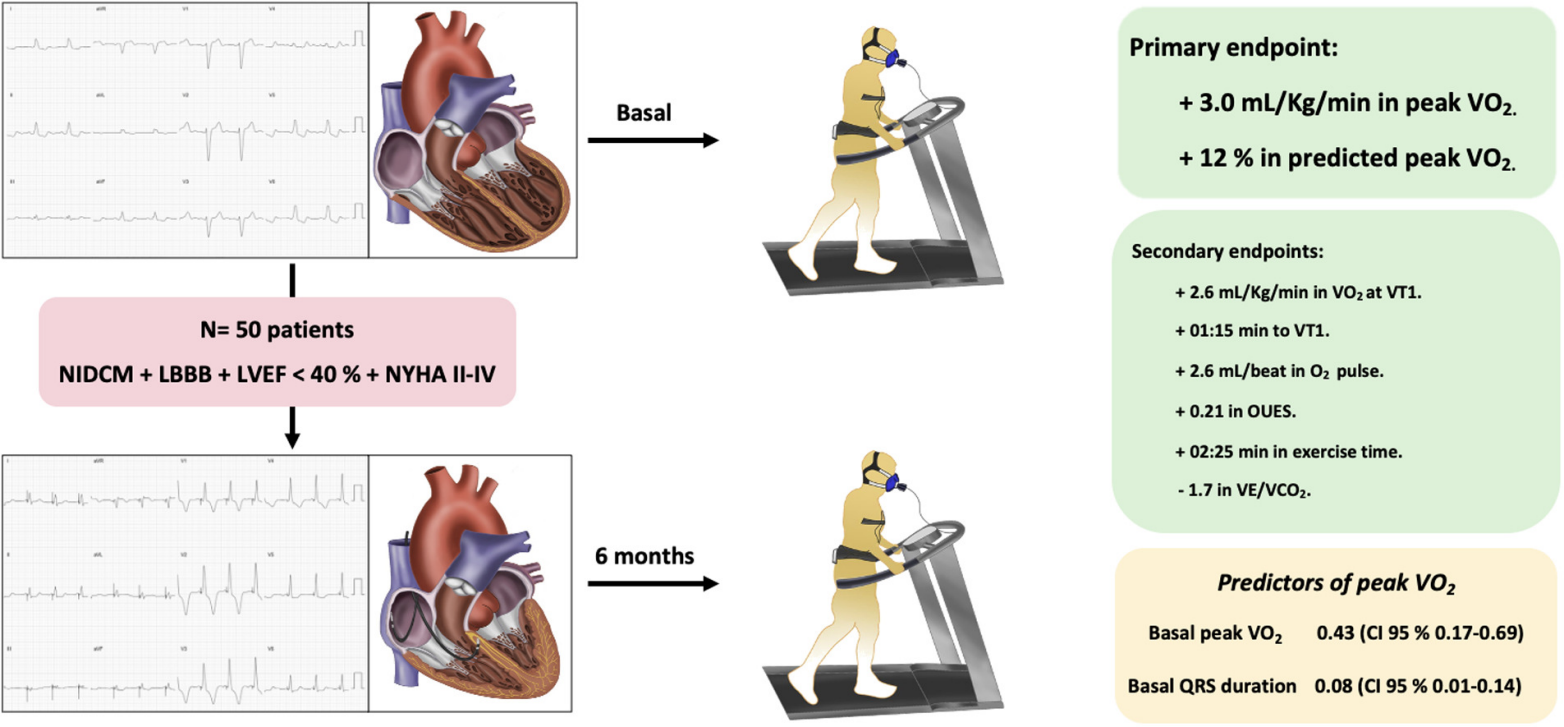
We included 50 patients with non-ischemic dilated cardiomyopathy, left bundle branch block (QRS >130 ms), NYHA functional class II–IV, and left ventricular ejection fraction <40% despite optimal medical treatment. All patients underwent baseline cardiopulmonary exercise testing and a follow-up test 6 months after left bundle branch area pacing. Within the primary endpoint, we observed an improvement in peak oxygen uptake of 3 ml/kg/min and an increase of 12% in predicted peak oxygen uptake. Among the secondary endpoints, we observed an improvement in oxygen uptake at the first ventilatory threshold as well as an increase in the time to reach it, oxygen pulse, OUES, exercise time, and VE/VCO_2_. In multivariate analysis, the predictors of peak oxygen uptake at follow-up were baseline QRS duration (ms) and baseline peak oxygen uptake. LBBB, left bundle branch block; LVEF, left ventricular ejection fraction; NIDCM, non-ischemic dilated cardiomyopathy; NYHA, New York Heart Association; OUES, oxygen uptake efficiency slope; VE/VCO_2_, minute ventilation–carbon dioxide production relationship; VO_2_, oxygen uptake; VT1, first ventilatory threshold.

**Table 3:** Cardiopulmonary exercise testing parameters.^a^

	Baseline	Follow-up	Changes in follow-up	*p*
Peak VO_2_ (ml/kg/min)	15.3 ± 4.9	18.3 ± 5	+3.0 (1.7–4.4)	<0.01^b^
Percent-predicted peak VO_2_ (%)	69 ± 20	81 ± 23	+12 (5–19)	<0.01^b^
RER	1.090 ± 0.16	1.107 ± 0.15	+0.017 (−0.04 to 0.07)	0.54^b^
Exercise time (min:s)	07:23 (05:38–10:25)	10:19 (07:35–13:15)	+ 02:25 (01:25–03:29)	<0.01^c^
VO_2_ at VT1 (ml/kg/min)	10.5 ± 2.9	13.1 ± 3.3	+2.6 (1.6–3.5)	<0.01^b^
Time to VT1 (min:s)	03:55 (02:40–04:45)	05:10 (03:50–07:20)	+01:15 (00:45–02:00)	<0.01^c^
Peak oxygen pulse (ml/beat)	9.6 ± 4.1	12.2 ± 4.4	+2.6 (1.5–3.7)	<0.01^b^
OUES	1.69 ± 0.69	1.91 ± 0.65	+0.21 (0.05–0.37)	<0.01^b^
VE/VCO_2_ slope	31.1 (27.4–34.6)	28.1 (25.4–33)	−1.7 (−3.6 to −0.2)	0.04^c^
		Baseline	Follow-up	*p*
Ventilatory class, *N* (%)	I	20 (40%)	33 (66%)	<0.01^d^
	II	23 (46%)	8 (16%)	
	III	4 (8%)	5 (10%)	
	IV	3 (6%)	4 (8%)	

OUES, oxygen uptake efficiency slope; RER, respiratory exchange ratio; VE/VCO_2_, minute ventilation–carbon dioxide production relationship; VO_2_, oxygen uptake; VT1, first ventilatory threshold.

^a^ Quantitative variables are expressed as mean ± standard deviation or as median and interquartile range as appropriate. Qualitative variables are expressed as absolute values and percentages. Variations in quantitative variables during follow-up are expressed as means with a 95% confidence interval.

^b^ Paired Student's *t*-test.

^c^ Wilcoxon test.

^d^ McNemar test.

### Predictors of peak VO_2_ response at follow-up

In the univariate analysis, predictors of peak VO_2_ at follow-up were younger age, absence of hypertension, baseline peak VO_2_, greater KCCQ-12 score, better renal function, higher ferritin values, better FC, longer 6MWT distance, greater left ventricular end-systolic volume (LVESV), greater left ventricular end-diastolic volume (LVEDV), longer baseline QRS, and paced QRS duration. In the multivariate analysis, the predictors of peak VO_2_ at follow-up were baseline peak VO_2_ (beta 0.43, 95% CI 0.17–0.69, *p* < 0.01) and baseline QRS duration (beta 0.08, 95% CI 0.01–0.14, *p* < 0.01) (Table 4).

**Table 4:** Predictors of peak oxygen uptake after left bundle branch area pacing. Univariate and multivariate linear regression models.

Variables	Univariate			Multivariate		
	Beta	95% CI	*p*	Beta	95% CI	*p*
Age (y.o)	**−0**.**21**	**−0.34 to −0.09**	**<0**.**01**			
HTN	**−4**.**58**	**−7.18 to −1.98**	**<0**.**01**			
DM	**−**2.36	**−**5.60 to 0.88	0.15			
Male sex	**−**1.51	−4.42 to 1.39	0.30			
BMI (kg/m^2^)	**−**0.01	−0.056 to 0.043	0.79			
Basal peak VO_2_ (ml/kg/min)	**0**.**54**	**0.29–0.80**	**<0**.**01**	**0**.**43**	**0.17–0.69**	**<0**.**01**
Basal KCCQ-12	**0**.**18**	**0.06–0.31**	**<0**.**01**			
Basal NT-ProBNP (pg/ml)	0.00	−0.00 to 0.00	0.39			
Basal creatinine (mg/dl)	**−5**.**25**	**−10.3 to −0.23**	**0**.**04**			
Basal ferritin (ng/ml)	**0**.**023**	**0.01–0.04**	**<0**.**01**			
Basal Haemoglobin (g/dl)	0.86	−0.22 to 1.94	0.12			
Basal 6MWT (m)	**0**.**03**	**0.02–0.05**	**<0**.**01**			
NYHA III vs. II	**−4**.**72**	**−7.94 to −1.50**	**<0**.**01**			
Basal LVEF	−0.05	−0.26 to 0.15	0.58			
Basal LVEDV (ml/m^2^)	**0**.**03**	**0.01–0.05**	**<0**.**01**			
Basal LVESV (ml/m^2^)	**0**.**04**	**0.01–0.06**	**<0**.**01**			
LA volume (ml)	−0.09	−0.21 to 0.021	0.11			
Basal QRS duration (ms)	**0**.**12**	**0.05–0.18**	**<0**.**01**	**0**.**08**	**0.01–0.14**	**0**.**02**
Paced QRS duration (ms)	**0**.**15**	**0.05–0.25**	**<0**.**01**			
RV_6_–RV_1_ (ms)	0.06	−0.02 to 0.13	0.12			
LVAT (ms)	−0.01	−0.10 to 0.09	0.96			

6MWT, 6-min walk test; BMI, body mass index; DM, diabetes mellitus; HTN, hypertension; KCCQ-12, Kansas City Cardiomyopathy Questionnaire; LA, left atrium; LVAT-V6, interval between the pacing spike and the peak of the R wave in V6; LVEDV, left ventricular end-diastolic volume; LVEF, left ventricular ejection fraction; LVESV, left ventricular end-systolic volume; NYHA, New York Heart Association; RV_6_–RV_1_ interpeak interval, interval (ms) between the peak of the R wave in V6 and the peak of the R wave in V1; VO_2_, oxygen uptake.

The predictors of peak oxygen consumption at follow-up in the univariate model are shown in bold. In the multivariate model, baseline QRS duration and peak oxygen consumption were predictors of peak oxygen consumption at follow-up.

### Clinical, analytical, and echocardiographic results

**Figure 1:**
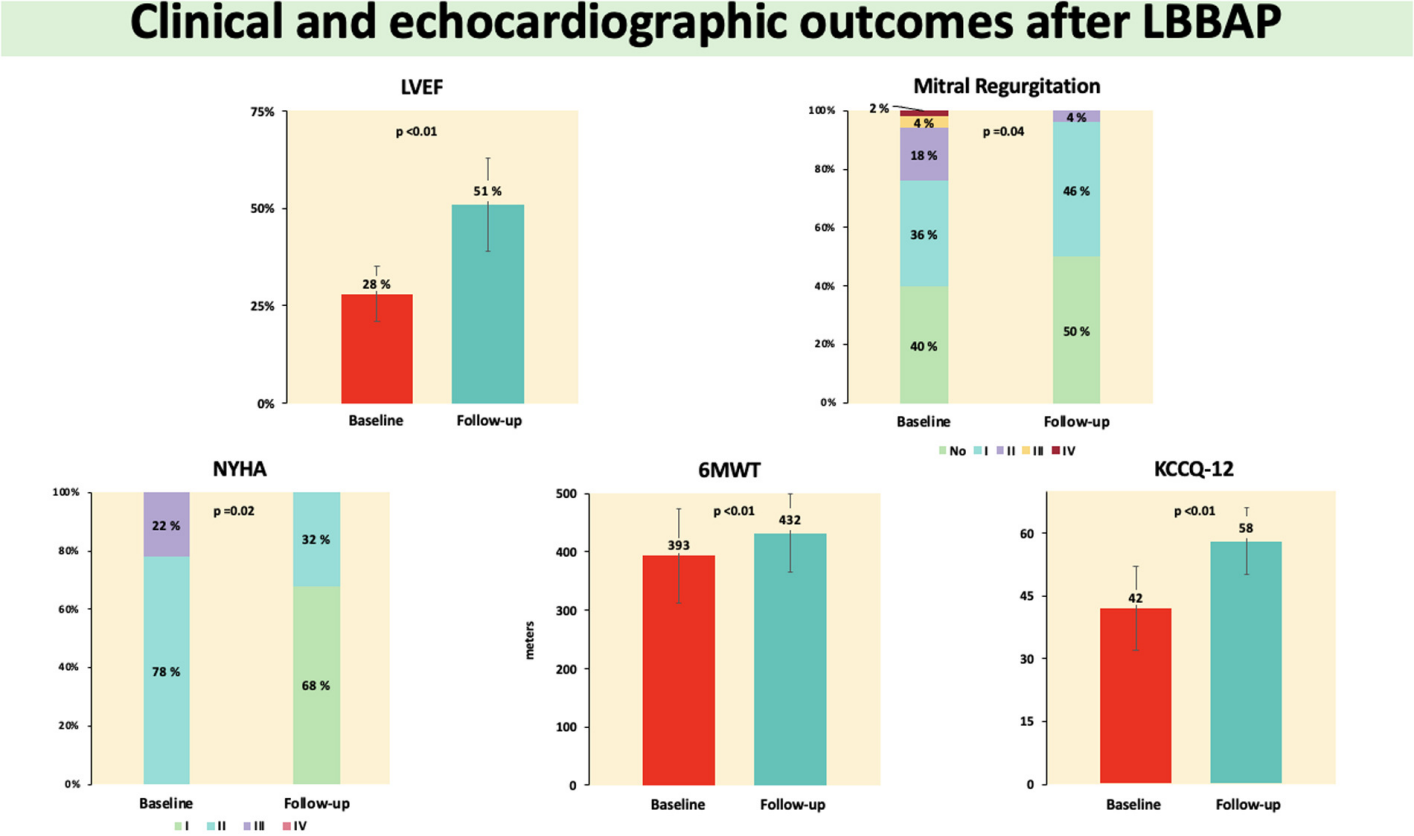
Outcomes in the main clinical and echocardiographic variables analyze quantitative variables are expressed as mean ± standard deviation or as median and interquartile range as appropriate. Qualitative variables are expressed as percentages. 6MWT, 6-min walk test; KCCQ-12, Kansas City Cardiomyopathy Questionnaire-12; LBBAP, left bundle branch area pacing; LVEF, left ventricular ejection fraction; NYHA, New York Heart Association.

An improvement in NYHA-FC after LBBAP was observed, such that at the end of follow-up, 34 (68%) patients were in NYHA-FC I and 16 (32%) in NYHA-FC II (*p* = 0.02). The distance walked in the 6MWT increased by 39 m (95% CI 19–60, *p* < 0.01) and the KCCQ-12 score by 11 points (95% CI 7–14, *p* < 0.01). In echocardiography, an absolute increase in LVEF of 23 points (95% CI 20–26, *p* < 0.01), a significant reduction in LVESV and LVEDV, and a lower number of patients with grade II–IV MR [12 (24%) patients at baseline vs. 2 (4%) patients at follow-up, *p* = 0.04] were observed. NT-ProBNP decreased at follow-up by 691 pg/ml (95% CI –1,060 to –393, *p* < 0.01) (Figure 1 and Table 5). No deaths were observed during follow-up; however, one patient had two admissions for heart failure during the study period.

**Table 5:** Clinical and echocardiographic outcomes.^a^

		Baseline	Follow-up	*p*
NYHA	I	0 (0%)	34 (68%)	0.02^b^
	II	39 (78%)	16 (32%)	
	III	11 (22%)	0 (0%)	
	IV	0 (0%)	0 (0%)	

	Baseline	Follow-up	Changes in follow-up	*p*
LVEF (%)	28 ± 7	51 ± 12	+23 (20–26)	<0.01^c^
LVEDV (ml/m^2^)	99 ± 31	57 ± 24	−42 (−31 to −51)	<0.01^c^
LVESV (ml/m^2^)	73 ± 28	31 ± 19	−42 (−33 to −51)	<0.01^c^

		Baseline	Follow-up	*p*
Mitral regurgitation	No	20 (40%)	25 (50%)	0.04^b^
	I	18 (36%)	23 (46%)	
	II	9 (18%)	2 (4%)	
	III	2 (4%)	0 (0%)	
	IV	1 (2%)	0 (0%)	

	Baseline	Follow-up	Changes in follow-up	*p*
6MWT (m)	393 ± 80	432 ± 67	+39 (19–60)	<0.01^c^
KCCQ-12 score	42 (35–58)	58 (49–63)	+11 (7–14)	<0.01^d^
NT-ProBNP (pg/ml)	1,075 (485–1,999)	254 (119–796)	−691 (−1,060 to −393)	<0.01^d^

6MWT, 6-min walk test; KCCQ-12, Kansas City Cardiomyopathy Questionnaire; LVEDV, left ventricular end-diastolic volume; LVEF, left ventricular ejection fraction; LVESV, left ventricular end-systolic volume; NYHA, New York Heart Association.

^a^ Quantitative variables are expressed as mean ± standard deviation or as median and interquartile range as appropriate. Qualitative variables are expressed as absolute values and percentages. Variations in quantitative variables during follow-up are expressed as means with a 95% confidence interval.

^b^ McNemar test.

^c^ Paired Student's *t*-test.

^d^ Wilcoxon test.

## Discussion

### Primary endpoint

The main finding of our study is the objective improvement in functional capacity in the population analyzed after LBBAP at 6 months of follow-up, as shown by the increase in peak VO_2_ measured with CET. The main predictors of peak VO_2_ in follow-up were baseline QRS duration and baseline peak VO_2_. In addition, improvement was observed in the rest of the CET, echocardiographic and clinical parameters, and NT-ProBNP levels.

This is the first study published that assesses the effects on VO_2_ in patients with NIDCM and indication for CRT after LBBAP. The evidence available to date shows improvement in clinical and echocardiographic parameters after LBBAP, both in observational and comparative studies with CRT-CS (5–8); however, there are no data related to changes in the different CET parameters. These parameters have prognostic importance, with peak VO_2_, both in absolute value and as a percentage of the predicted value, being the most used due to its simplicity of calculation, strong ability to predict (along with exercise time) cardiovascular events, and reproducibility (9). Other values such as VO_2_ at VT1, time to VT1, OUES, exercise time, O_2_ pulse, or ventilatory class have also been shown to be predictors of mortality, need for heart transplantation, and hospitalizations for heart failure during follow-up (9, 12, 20, 21). Furthermore, peak VO_2_ not only has prognostic significance, but it is also directly related to the patients' quality of life (22).

Our study shows a significant improvement in peak VO_2_ (+3.0 ml/kg/min) and in %ppVO_2_ (+12%) in the population analyzed. The patients enrolled had a severely depressed LVEF (28% ± 7%) and were in adequate OMT. Additionally, 78% were in NYHA-FC II, the median score on the KCCQ-12 was 42 points, and, on average, according to peak VO_2_ achieved in baseline CET, they were in Webber class C (23); therefore, despite being a population with an advanced degree of heart disease, although with non-limiting symptoms derived from heart failure, CRT with LBBAP increased not only peak VO_2_ but also improved others maximal and submaximal variables with prognostic character: VO_2_ at VT1 along with an increase in the time to reach it, O_2_ pulse, VE/VCO_2_ *slope*, and OUES, as well as the percentage of patients in ventilatory class I at the end of follow-up (Table 3). Moreover, the increase in peak VO_2_ determined that at the end of the study, the patients were on average within Webber class B, with the prognostic improvement that this entails (20, 21, 23). No differences were observed in RER, but this parameter has not demonstrated prognostic value (9). The increase in peak VO_2_ of 3 ml/kg/min and 12% of %ppVO_2_ observed in our study has an important prognostic impact as observed by Keteyian et al. (9), who described, in a cohort of 2,100 patients with heart failure, that each reduction in peak VO_2_ of 1 ml/kg/min or 5% of %ppVO_2_ was associated with a 16% and 19% increase in mortality, respectively. In our population, CPET was maximal in 58% at baseline and 50% at follow-up with no significant differences between them (*p* = 0.15). Although this fact prevents us from obtaining the maximum VO_2_ in a significant number of patients, it does not limit the value of the results obtained because, according to what was observed by Keteyian et al., the predictive value of peak VO_2_ and exercise time to discriminate mortality is similar at RER values >0.95, which implies that in CET with RER between 0.95 and 1.1, the predictive value of these parameters remains valid (9).

If we analyze the data published on improvement in peak VO_2_ determined by CRT-SC, we observe that the increase ranges from +1.1 to +3.0 ml/kg/min, while VT1 varies between +1.52 and +2.0 ml/kg/min, data similar to those obtained in our study (19, 24, 25). Despite not being comparable populations, our results add new information to the available evidence to support LBBAP being considered an alternative to CRT-CS. For the other parameters assessed in CET that showed improvement after LBBAP (OUES, O_2_ pulse, VE/VCO_2_ slope, ventilatory class), data after CRT-CS are limited.

As for predictors of peak VO_2_ at follow-up, the only significant variable in the multivariable model (in addition to baseline peak VO_2_) was baseline QRS duration, not QRS narrowing or final QRS duration. These results suggest that after successfully implanting an LBBAP device, the degree of improvement in peak VO_2_ experienced by the patient depends primarily on baseline QRS duration. Although no predictors of peak VO_2_ response have been evaluated with CRT-CS, predictors of echocardiographic response in NIDCM have been described, including female sex, lower body mass index, and smaller left atrial size; in our population, these parameters were not predictors of peak VO_2_ at follow-up (Table 4) (26).

### Secondary endpoints

In the secondary endpoints, we observed an increase in LVEF of 23 points, a significant decrease in LVEDV and LVESV, and a reduction in the severity of MR, with similar results to those reported by other groups (5–8). These data were accompanied by a significant reduction in NT-ProBNP levels and a significant and clinically relevant increase of 39 m in 6MWT (27). The changes observed in CET and ventricular remodeling parameters resulted in an improvement in NYHA-FC at follow-up, such that at the end of the study 68% were in FC I, with an increase of 11 points on the KCCQ-12, which represents a moderate-high improvement in patients' quality of life, with the consequent clinical, prognostic, and psychological benefit (22). Finally, device implantation was successful in 98% of cases, with adequate pacing and detection parameters both acutely and at follow-up and a low percentage of complications (one acute lead dislocation and onr threshold increase of 0.75 V without LBBAP compromise), which means, on the one hand, that it is a safe and reproducible technique and, on the other hand, that it is an efficient technique, both in terms of the type of device implanted (dual-chamber compared with biventricular in the case of CRT-CS implantation) and energy consumption (mean threshold at follow-up of 0.56 ± 0.17 V/0.4 ms), in contrast to the data published in recent series where the mean stimulation threshold of the CS lead was >1 V (7, 8).

## Limitations

This study has several limitations. First, the results obtained are not applicable to other groups of patients with an indication for CRT, such as those with ischemic DCM or DCM secondary to chronic RV pacing. Second, the single-center nature of the study and the limited sample size may limit the extrapolation of the results obtained. Third, since the study's follow-up period was 6 months, it is unknown whether the results are maintained, improved, or worsened over the long term. Another limitation is the possible placebo effect that the implantation of the device may have on the patient, increasing motivation to perform the control CET with the consequent increase in peak VO_2_; however, the significant increase of a similar magnitude in the values obtained in parameters not dependent on the patient's motivation to perform the CET (VO_2_ at VT1 and time to VT1) appears to reduce the influence of the possible placebo effect on the results. Although the OMT of the study population was adequate, the rate of ISGLT-2 prescribed was slightly lower than that of other HF drugs, and whether this may have an impact on the results obtained is unknown. Regarding cardiac imaging parameters, although we observed an improvement in LVEF, ventricular volumes, and mitral regurgitation severity in TTE, global longitudinal strain analysis was not included in the study protocol, and it could provide additional information, in the same way, cardiac MRI was only recommended in young patients to assess LGE and evaluate concomitant ICD implantation, which is also a limitation since its routine use could add diagnostic and prognostic value.

Finally, the absence of a control group with CRT-CS does not allow for comparison of results with the therapy currently considered first-line in the study population.

## Conclusion

In symptomatic NIDCM and LBBB patients, LBBAP was a safe procedure with a high success rate and was associated with a significant and clinically relevant increase in peak VO_2_. Both baseline peak VO_2_ and baseline QRS duration were predictors of peak VO_2_ at follow-up after LBBAP.

## Data Availability

The original contributions presented in the study are included in the article/Supplementary Material; further inquiries can be directed to the corresponding author.
